# Study on Optimization of Drilling Parameters for Laminated Composite Materials

**DOI:** 10.3390/ma16051796

**Published:** 2023-02-22

**Authors:** Jiali Yu, Tao Chen, Yiming Zhao

**Affiliations:** 1 State-Owned Wuhu Machinery Factory, Wuhu 241000, China; 2 School of Future Science and Engineering, Soochow University, Suzhou 215299, China

**Keywords:** composite, delamination inhibition, processing parameters

## Abstract

Fiber-reinforced resin matrix composites have been widely used in aerospace, construction, transportation and other industries due to their excellent mechanical properties and flexible structural design. However, due to the influence of the molding process, the composites are easily delaminated, which greatly reduces the structural stiffness of the components. This is a common problem in the processing of fiber-reinforced composite components. In this paper, through the combination of finite element simulation analysis and experimental research, drilling parameter analysis was carried out for prefabricated laminated composites, and the influence of different processing parameters on the processing axial force was qualitatively compared. The inhibition rule of variable parameter drilling on the damage propagation of initial laminated drilling was explored, which further improves the drilling connection quality of composite panels with laminated materials.

## 1. Introduction

Carbon fiber-reinforced resin matrix composite components are often designed and manufactured by laying, curing, processing and assembly, which is widely used in aerospace, automobile, rail transit marine structure and other fields [1,2]. However, due to the different physical and chemical properties of each constituent phase of carbon fiber composites, fiber wrinkling, delamination, porosity and other defects often occur due to an uneven resin flow at the bending part of the component, unsynchronized curing of the internal and external surfaces, foreign matters entering and other factors during the curing process [3,4,5]. Among them, delamination defect is the most frequently forming defect, whose distribution is random and uncontrollable, which significantly reduces the interlayer bonding strength of the components and seriously affects the mechanical properties of the components [6,7,8,9]. The composite main load-bearing component is the core component of aircraft equipment, which bears huge loads and has a complex service environment [10,11,12,13]. In order to ensure its high load-bearing performance and service reliability, the processing accuracy of the component is strictly controlled. In order to achieve the required surface quality, geometric accuracy and some special functional requirements of the parts, it is inevitably required to carry out a series of cuttings on the cured components. Once the layering defect expands, a single expensive component will be scrapped, to prevent a serious safety accident [14]. Therefore, it is necessary to effectively restrain the propagation of the formation of delamination defects during drilling. Many scholars have also carried out relevant research on the drilling mechanism, process and assembly of composite panel structures [15,16,17].

Luo et al. put polytetrafluoroethylene into a glass fiber epoxy resin composite to simulate the molding delamination defect during the preparation of the composite, and carried out compression tests on it to study and analyze its delamination mode [18]. The results showed that it was reliable to conduct tests with the delamination defect embedded in the polytetrafluoroethylene film. Pekbey and Sayman et al. studied the critical buckling load of glass fiber composite laminates with zonal delamination damage, and found that the fiber ply angle, ply sequence and specimen size all affect the critical buckling load [19]. J. Xiong et al. studied the formation and evolution of the delamination damage of composite laminates with holes under fatigue compression load, and pointed out that the stiffness changes little during the formation of delamination damage [20]. M. Kenane et al. carried out static load and fatigue load tests on composite laminates and measured the delamination fatigue growth threshold [21]. However, the actual drilling process is a dynamic process under the continuous feed of the tool. The size of the drilling force is not constant and the position of the action point changes dynamically with the rotation and feed movement of the tool edge. Arman et al. studied the influence of a single circular delamination around a circular hole on the mechanical properties of material laminated plates, pointing out that when the delamination diameter reaches a certain value, the critical buckling load value decreases rapidly, and the fiber direction also has an influence on the critical load value [22].

Because there is a certain mathematical relationship between the drilling process parameters of fiber reinforced composites and the drilling axial force, it is possible to control the drilling process parameters so that the axial force does not exceed the critical value and thus suppress the delamination defects (mainly the exit delamination defects). Many scholars have done a lot of research on the mathematical relationship between the axial force and the process parameters in the drilling process of fiber-reinforced composites. Most of the mathematical relationships between drilling process parameters and axial force are empirical curve fitting expressions based on experimental data. The axial force variables investigated are spindle speed, feed rate, drill tip angle, or tool wear [23,24,25]. Similarly, some scholars obtained the critical drilling parameters corresponding to different drilling residual material thicknesses through experiments, and realized the exit delamination defect suppression in the multi-stage variable parameter drilling process [26,27,28].

Although the related research has analyzed the rule and process of composite delamination damage, there is still no clear result on how to design the relevant processing parameters for laminates with delamination. Therefore, composite laminates with delamination defects are prefabricated in the paper. Through finite element simulation and experimental verification, the influence of different processing parameters on the processing quality is analyzed, and a variable parameter processing method is proposed, which provides a scientific basis for the optimization of the processing parameters of composites with delamination.

## 2. Experiment and Simulation

### 2.1. Laminate Sample

**Figure 1:**
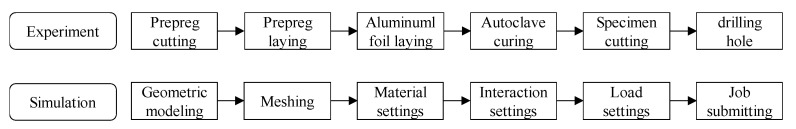
Scheme of experiment and simulation.

In this paper, T3000/BA9913 prepreg is used for sample preparation, as follows. First, take the prepreg out of the refrigerator and warm it at room temperature for more than 6 h. Lay according to the angle sequence of [45/-45/0/-45/0/-45/0/-45/90/45/-45/0] s. When laying 12 to 13 or 18 to 19 layers of prepreg, 90 mm long axis and 30 mm short axis oval shaped aluminum foils are placed. In this way, prefabricated delamination defects at 1/2 or 3/4 thickness could be obtained in order to simulate the actual delamination damage. Then, put the prepreg with aluminum foils into the autoclave, heat at 3 °C/min to 120 °C, cure at 0.6 MPa for 2 h, and finally cool to 60 °C at a rate of 1.5 °C/min. After that, cut the laminate into 160 × 160 mm size specimens using a CNC machine tool. The scheme of experimental and numerical parts is shown in Figure 1.

### 2.2. Finite Element Simulation Model

ABAQUS 6.14 was used for finite element modeling, including composite laminates and drill bits. The geometric size and ply angle of the composite material were consistent with the test sample. C3D8R grid was adopted, with the overall size of 2 mm and the grid size of the layered area of 1 mm. The CATIA V5R21 software was applied to draw the model according to the actual shape of the drill. The HyperMesh was used to divide and mesh the drill model and imported into ABAQUS through the interactive interface. Finally, the model was repaired to reduce the non-convergence caused by the model accuracy. To simulate the delamination defect in the middle of the laminated plate, the mesh offset function was used to set a cohesive grid element of 0 thickness at the delamination of the test piece. In order to ensure the convergence of the model and reduce the amount of calculation, the central area of the workpiece, the cross edge of the drill bit and the main cutting edge were refined.

In the material property setting, the drill bit was made of high-speed steel, with an elastic modulus of 200 GPa, Poisson’s ratio of 0.3 and a density of 6.7 g/cm^3^. The material parameters of T300/BA9913 composite laminate are shown in Table 1.

The interaction between the tool and the workpiece was set as tangential friction contact, normal hard contact, and the friction coefficient between the drill and the workpiece was set as 0.15. All six degrees of freedom of the composite plate were constrained to ensure that the workpiece does not move during drilling.

**Figure 2:**
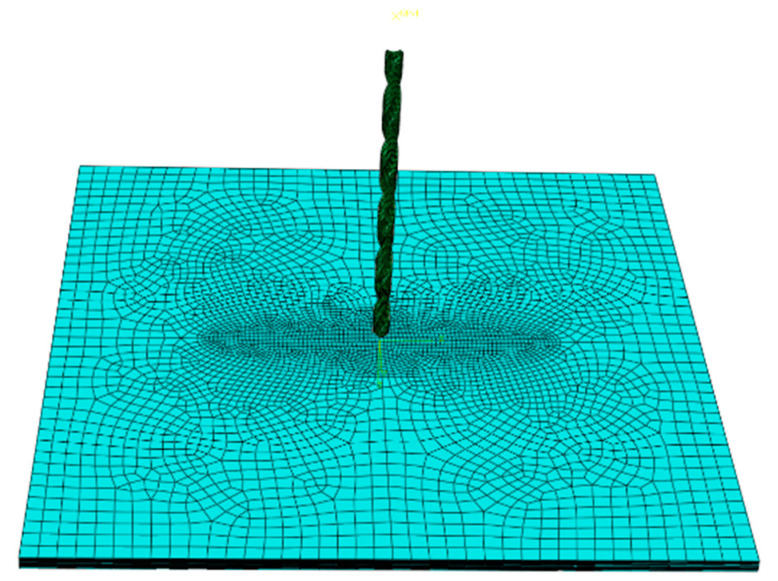
Finite Element Analysis Model.

In order to effectively apply the processing load of the drill bit, a reference point was set outside the drill bit along the direction of the drill bit’s central axis. The coupling constraint was applied to the reference point and the upper end face of the drill bit to realize synchronous load transfer between them. After that, the feed rate along the central axis of the drill bit and the rotation speed around the central axis of the drill bit were applied to the reference point. All other degrees of freedom were constrained. Thus, the drilling load could be applied. The final model is shown in Figure 2.

The mesh type of the laminate was C3D8R, the drill was assigned as rigid, and the pre-delamination was assigned as cohesive mesh. The size of the central area mesh was 0.5 mm. Hashin damage criterion was adopted for laminate mesh, and the failure criteria are expressed as follows.

Fiber tensile failure ($\sigma_{11}>0$),

(1) $$F_{T}={\left(\frac{\sigma_{11}}{X_{T}}\right)}^{2}+\alpha {\left(\frac{\tau_{12}}{S_{12}}\right)}^{2}$$

Fiber compressive failure ($\sigma_{11}<0$),

(2) $$F_{C}={\left(\frac{\sigma_{11}}{X_{C}}\right)}^{2}$$

Matrix tensile failure ($\sigma_{22}>0$),

(3) $$M_{T}={\left(\frac{\sigma_{22}}{Y_{T}}\right)}^{2}+\alpha {\left(\frac{\tau_{12}}{S_{12}}\right)}^{2}$$

Matrix compressive failure ($\sigma_{22}<0$),

(4) $$M_{C}={\left(\frac{\sigma_{22}}{2S_{21}}\right)}^{2}+\left[{\left(\frac{Y_{C}}{2S_{21}}\right)}^{2}\right]\frac{\sigma_{22}}{Y_{C}}+{\left(\frac{\tau_{12}}{S_{12}}\right)}^{2}$$

$\sigma_{11}$, Longitudinal stress; $\sigma_{22}$, Transverse stress; $F_{T}$, Fiber tension damage; $F_{C}$, Fiber compression damage; $M_{T}$, Matrix tension damage; $M_{T}$, Matrix compression damage; $\tau_{12}$, Shear stress; $S_{12}$, Longitudinal shear strength; $S_{21}$, Transverse shear strength; $X_{T}$, Longitudinal tensile strength; $X_{C}$, Longitudinal compressive strength; $Y_{T}$, Transverse tensile strength; $Y_{C}$, Transverse compressive strength.

Dynamic analysis step was adopted, and F-Output-1 was created in the field output manager, the action area was set as the whole model, and the time interval was 40 incremental steps. The fiber damage, matrix damage, fiber tensile damage, fiber compression damage, matrix tensile damage, matrix compression damage, shear damage and damage initial criterion were selected in the output variables, and finally the output variable status to set to STATUS.

### 2.3. Drilling Processing Test

XK7124 CNC vertical bed drilling machine (manufactured by ZOJE CNC Machine Tool Co., Ltd. No. 28-14, Shenying Road, Hunnan District, Shenyang, China) was used for drilling processing test. The force measuring instrument used in the test was 9129AA multi-component force measuring instrument produced by Kistler Company(Building 15, Lane 1588, Shenchang Road, Minhang District, Shanghai, China). The test processing tool was 4.05 mm carbide steel drill (Sandvik Company, Room JT15049, No. 912, Yecheng Road, Jiading Industrial Zone, Shanghai, China). According to the test design matrix of drilling parameters, each group of drilling processing samples was at least 3.

**Figure 3:**
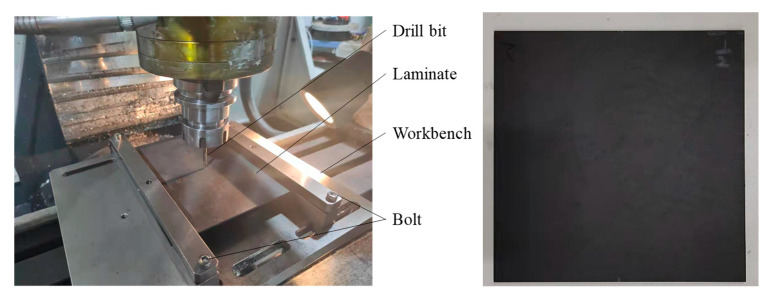
Test Equipment and sample.

During the drilling experiment, the piezoelectric effect was generated by the force acting on the piezoelectric chip of the Kistler 9327B dynamometer, and then the electrical signal was amplified by the Kistler 5073A charge amplifier. The signal was collected by the Kistler 5697A data acquisition card with a sampling frequency of 20 KHz. The signal was transmitted to the computer for post-processing. Use Dynoware 2825A software(Building 15, Lane 1588, Shenchang Road, Minhang District, Shanghai, China) to filter and analyze the collected force signal. The experimental apparatus and sample were shown in Figure 3.

## 3. Results and Analysis

In view of the increase of cutting force caused by the layer thickness and rotating speed, variable parameter drilling control tests were carried out. In this paper, different processing parameters were taken before and after the pre layering. The specific test design matrix is shown in Table 2.

### 3.1. The Influence of Constant and Dynamic Feed Speed

**Figure 4:**
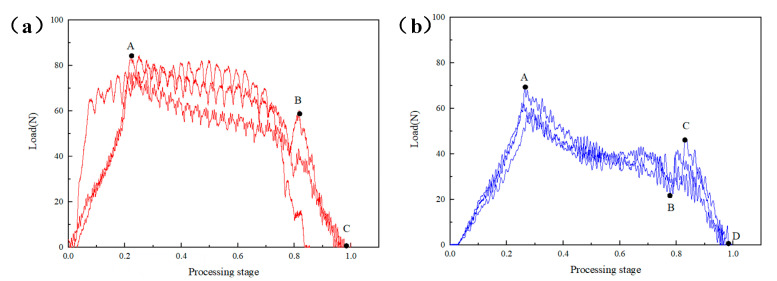
Axial force time curves of constant and dynamic feed speeds with rotation speed of 4000 r/min: (**a**) Constant feed speed 10 mm/min, (**b**) Dynamic feed speed 5–10 mm/min.

The sample was a 3/4 thick prefabricated laminated composite material sample. During the processing at the same speed of 4000 rpm, the effects of the constant feed speed and the low-speed high-speed dynamic feed speed on the axial force of the drill bit were compared. Figure 4a shows the axial force time curve at a constant feed speed of 10 mm/min. The processing process can be divided into three stages. The first stage was the O-A stage. At this time, the drill bit had just started to contact the laminate, the cutting edge of the drill bit was cutting composites, the contact area between the cutting edge and the laminate was increasing and the longitudinal force was gradually increasing from zero until the cutting edge at point A completely penetrated the laminate, with the maximum load of 84.38 N, followed by the A-B stage. The drill bit gradually drilled into the laminate, and the residual thickness gradually decreased with the constant feed of the drill bit. The longitudinal cutting force decreased slowly until point B; when the drill bit was about to drill through the laminate, the drilling force dropped rapidly until the end point C.

Figure 4b shows the feed speed of 5 mm/min for the first 1/2 thickness and 10 mm/min for the last 1/2 thickness. Compared with Figure 4a, it shows several differences. First, the curve slope of OA section was lower, and it was only 68.55 N compared with the maximum load value in Figure 4a. This was because the drill bit contacts gently and the cutting force was smaller during the processing of the first 1/2 thickness due to the slow feed; second, after point B, the cutting force had a short rise, that is, segment BC. This was because, after point B, the feed speed suddenly increased, and the resistance increased in the longitudinal feed process of the drill bit. The CD segment after point B was the same as the constant processing speed. Therefore, the total axial processing load of the laminate can be reduced to a certain extent by controlling the penetration rate so as to obtain better processing quality.

### 3.2. Effect of Constant and Dynamic Rotation Speed

**Figure 5:**
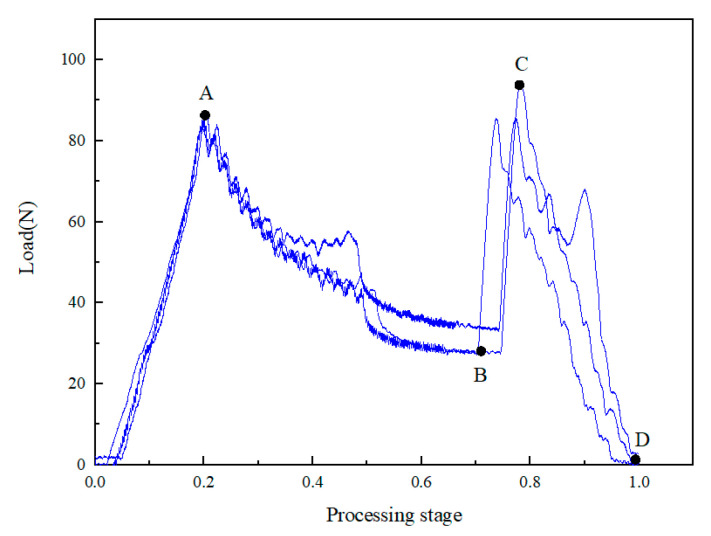
Axial force time curve of composites at dynamic rotation speed 4000–2000 r/min.

With the same feed speed of 10 mm/min, the influence of the constant speed and the high-speed low-speed dynamic speed on the axial force of the drill bit was compared. Figure 5 shows the axial force time curve during drilling with a variable speed of 4000–2000 r/min. Compared with Figure 4a, the results show that the BC segment of the variable speed machining was more significant, and its slope was far greater. It is noteworthy that the maximum load at point C reaches 91.25 N, which was very close to point A. This is because when the drill was processed to 3/4 thickness, the rotation speed decreased and the cutting resistance of the drill increased. Therefore, the influence of the rotational speed on delamination is likely to be greater than the advance speed for the 3/4 thick prefabricated layered samples. With reference to the 3/4 thick prefabricated layered samples, further verification tests of 1/2 thick prefabricated layered samples were carried out.

### 3.3. The Influence of Constant and Dynamic Feed Speed

The sample was a 1/2 thick prefabricated laminated composite sample. During the processing at the same speed of 4000 rpm, the effects of the constant feed speed and high-speed low-speed dynamic feed speed on the axial force of the drill bit were compared.

**Figure 6:**
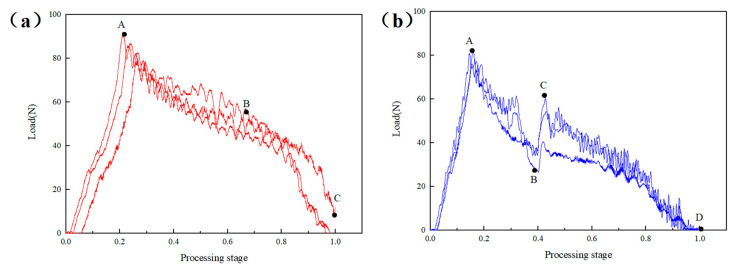
Axial force time curves of constant and dynamic feed speeds with rotation speed of 4000 r/min: (**a**) Constant feed speed 10 mm/min, (**b**) Dynamic feed speed 10–5 mm/min.

Figure 6a showed the sample processed at the feed speed of 10 mm/min, and its overall load curve was similar to Figure 5a. The difference was that the slope of section AB of 1/2 thick prefabricated layered sample was greater than 3/4 thick, and the slope of section BC was less than 3/4 thick. This was because the residual machining thickness of the 1/2 thickness sample was greater than that of the 3/4 thickness sample when it was finally drilled out, and the drill needed to overcome greater resistance to complete the cutting. The same rule was shown in the CD section of the sample processed at a variable speed, but the C point load was less than the C point load in Figure 5, indicating that the dynamic adjustment of the high feed speed low feed speed had a better effect on reducing the axial force of the drill bit.

### 3.4. The Influence of Constant and Dynamic Rotation Speed of 1/2 Layered Sample

In the process of machining with the same feed speed of 10 mm/min, the influence of the constant speed and low-speed high-speed dynamic speed on the axial force of the drill bit was compared.

**Figure 7:**
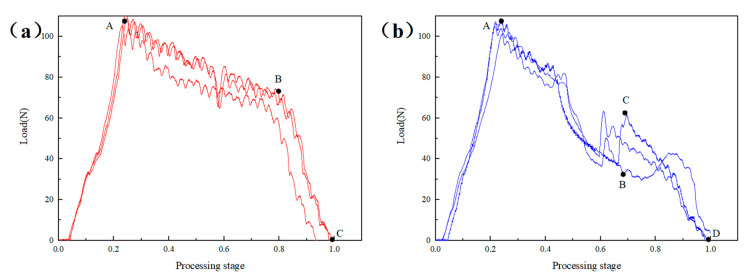
Axial force time curve of composites at constant and dynamic rotations speed: (**a**) Constant speed 2000 r/min, (**b**) Dynamic speed 2000–4000 r/min.

Figure 7 shows the axial force time curve during drilling with a constant speed of 4000 rpm and a variable speed of 4000 to 2000 r/min. The results show that, at the initial stage of the OA section at three speeds, slope fluctuation occurred, which was related to the instability of the contact force between the drill bit and the laminate at the beginning, but on the whole, it increased slowly and linearly with time. Compared with Figure 6a, for the 2000 r/min and 4000 r/min processing models, the curve characteristics of section AB and section BC were relatively close. In general, A and B of the 2000 r/min processing models were greater than the 4000 r/min. It is worth noting that no matter what speed was used for processing, the cutting force fluctuated at 1/2 of the processing stage. The same feature can also be observed in the 1/2 thickness prefabricated layered variable feed speed machining curve, which was caused by the drill bit starting to process the layered area at this time.

In the drilling axial force time curve of variable speed 4000–2000 r/min, the axial cutting force rising stage of BC segment also appeared. However, the load at point C was only 63.47 N, about 58% of the maximum load at section AB, which was far less than the load at point C in Figure 6, indicating that the dynamic adjustment from low speed to high speed had a better effect on reducing the axial force of the drill bit.

### 3.5. Finite Element Analysis of Mesh Damage

**Figure 8:**
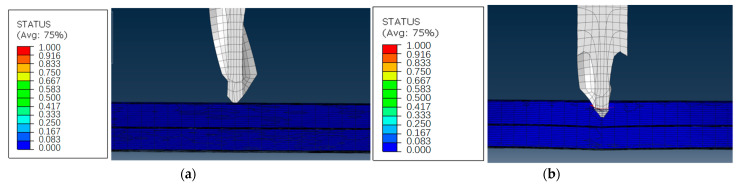

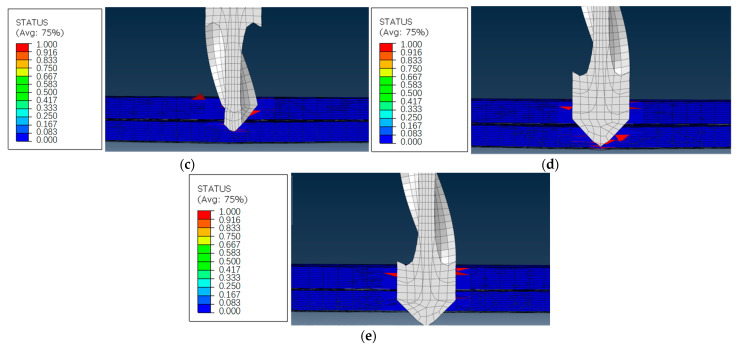
Drilling Stages: (**a**) Initial stage, (**b**) Blade feed, (**c**) Delamination cutting, (**d**) Critical stage, (**e**) Drilling out stage.

The processing stage can be divided into several parts, as shown in Figure 8a. This was the point O of the curve. The drill bit contacted the laminate at the beginning of the initial analysis step. Then, it entered the cutting stage, as shown in Figure 8b. The blade continuously entered the laminate, and the laminate bent downward under the longitudinal feed of the tool, causing tensile and compressive damage to the fibers and matrix near the cutting edge. With the gradual feed of the drill bit, the drill bit reached the 1/2 delamination interface. At this time, the upper and lower interfaces opened, and there was obvious delamination near the drill bit. As shown in Figure 8c, the longitudinal feed load reached a new peak. After that, the remaining cutting materials under the drill bit gradually reduced, and the feed load also decreased. The drill tip drills through the entire laminate, as shown in Figure 8d. Then the feed load dropped rapidly until the whole drill bit passes through the laminated plate, and the cutting process was completed, as shown in Figure 8e.

**Figure 9:**
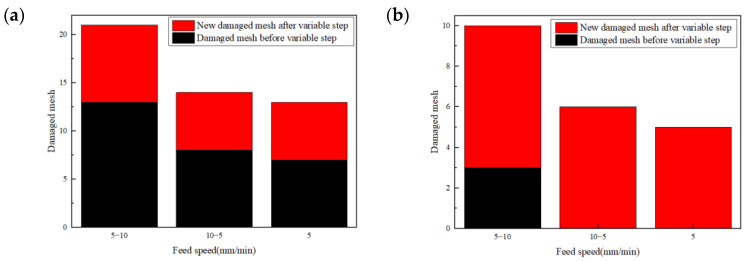
Mesh damage comparison before and after the change of process parameters in variable parameter analysis step: (**a**) laminated upper layer, (**b**) laminated lower layer.

**Figure 10:**
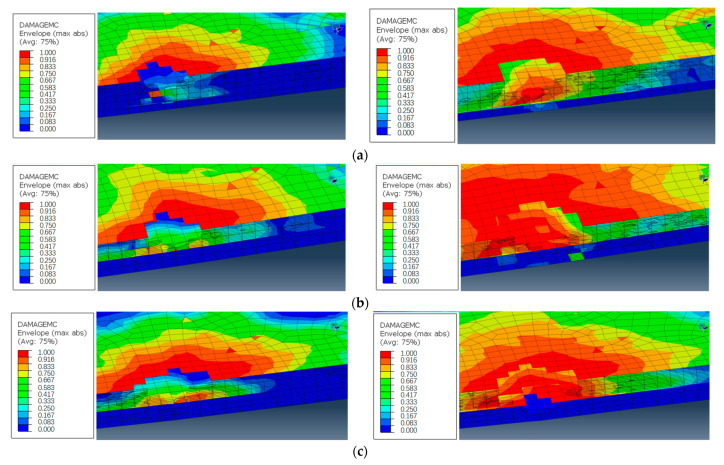
Hole wall mesh damage under different process parameters: (**a**) 5−10 mm/min, (**b**) 10−5 mm/min, (**c**) 5 mm/min.

Furthermore, this paper compares the damage of the lower platen at different emergency speeds: the damage of the feed speeds of 5–10 mm/min, 10–5 mm/min and the constant 5 mm/min machined samples before and after the variable parameter analysis step, as shown in Figure 9a,b, respectively. Before the step of the variable parameter analysis, the mesh damage of the hole wall of the laminated plate above the prefabricated delamination caused by the high feed speed was relatively large. At the same time, because the longitudinal feed speed was too fast, the laminated plate below the 5 mm/min processing sample also had a little mesh damage, while the 10 mm/min and variable speed processing sample did not have damage at this time, as is shown in Figure 10. High feed speed low feed speed machining was helpful to reduce the damage.

## 4. Conclusions

In the paper, the processing parameters of carbon fiber-reinforced resin matrix composite laminates with initial delamination are studied, and the processing experiment analysis is carried out with the axial force as the judgment index. The finite element model is established and analyzed through ABAQUS, and the influence rules of different processing parameters on the drilling quality are compared. The following conclusions are drawn:

(1) For composite laminates with delamination, when the drill bit is fed to the delamination interface, it will cause the lower laminate to bend downward, resulting in an increase in axial force and hole wall damage in the subsequent processing.
(2) Different advance speeds, rotational speeds and delamination thicknesses have different effects on the axial force. The location of the delamination thickness would lead to sudden changes in axial force at different times in the processing process. The decrease in rotational speed and the increase in advance speed would lead to sudden change of axial force.
(3) When the drill bit is close to the original delamination position, adjust the process parameters according to the delamination situation of the component, which is helpful in reducing the longitudinal cutting force, inhibiting the hole wall damage in the delamination area, and achieving good processing results. In particular, the low speed high speed and high feed low feed processing forms can reduce secondary damage, which will greatly improve the drilling quality of the composite wallboard with delamination.

## Figures and Tables

**Table 1:** Performance Parameters of T300/BA9913 Composite.

**E_1_** **(GPa)**	**E_2_** **(GPa)**	**G_12_** **(GPa)**	**G_13_** **(GPa)**	**X_T_** **(MPa)**
135	8.8	4.47	4.47	1548
**X_C_** **(MPa)**	**Y_T_** **(MPa)**	**Y_C_** **(MPa)**	**S_12_** **(MPa)**	**S_23_** **(MPa)**
1426	55.2	218	80	68

**Table 2:** Variable parameter drilling design matrix.

Serial	Rotation Speed (r/min)	Feed Speed (mm/min)
1	4000	10
2	4000	5–10
3	4000	10–5
4	4000–2000	10
5	2000–4000	10

## Data Availability

No new data were created.
